# Identification of risk factors for postoperative delirium in elderly patients with hip fractures by a risk stratification index model: A retrospective study

**DOI:** 10.1002/brb3.2420

**Published:** 2021-11-22

**Authors:** Ye Wang, Lin Zhao, Changsheng Zhang, Qi An, Qianqian Guo, Jie Geng, Zhenggang Guo, Zhengpeng Guan

**Affiliations:** ^1^ The Department of Anesthesiology Peking University Shougang Hospital Beijing China; ^2^ The Anesthesia and Operation Center The First Medical Center The Medical School of Chinese PLA Beijing China; ^3^ The Department of Orthopedics Peking University Shougang Hospital Beijing China

**Keywords:** health services for the elderly, hip fractures, postoperative cognitive complications, risk assessment, risk factors

## Abstract

**Introduction:**

Postoperative delirium is one of the most common and dangerous psychiatric complications after hip surgery. The aim of this study was to investigate the incidence of postoperative delirium in elderly patients after hip fracture surgery and to identify risk factors for such, as part of developing a risk stratification index (RSI) system to predict a patient's risk of postoperative delirium.

**Methods:**

Elderly patients (aged 65 years or older) with hip fractures who had received surgical treatment in our hospital between March 2018 and December 2019 were retrospectively included. Clinical data were collected, and multivariate logistic regression analysis was performed to investigate the relevant risk factors of postoperative delirium. An RSI system was developed based on factors identified in the regression analysis.

**Results:**

Of 272 patients included, 52 (19.12%) experienced postoperative delirium. Drinking history (> 3/ week), the perioperative lactic acid level (Lac > 2 mmol/L), postoperative visual analog score (VAS) > 3, American Society of Anesthesiologists (ASA) physical status > II, application of the bispectral index, and preoperative diabetes were independent risk factors of postoperative delirium. When RSI ≥ 5, the rate of postoperative delirium significantly increased (*p* < .05).

**Conclusion:**

The RSI system developed here can safely guide postoperative outcomes of elderly patients with hip fractures, and RSI ≥ 5 may be able to predict the onset of postoperative delirium.

## Graphical abstract

This study investigated the incidence of postoperative delirium in elderly patients after hip fracture surgery, and to identify risk factors for such, as part of developing a risk stratification index (RSI) system to predict a patient's risk of postoperative delirium.

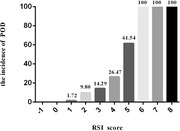

## 1 INTRODUCTION

Postoperative delirium (POD) is a common neurological complication that occurs following various surgical procedures (Rudolph & Marcantonio, 2011; van Meenen et al., 2014). POD is a serious complication because delirium leads to the worsening of patients’ prognosis, such as intolerance to tracheal intubation, a prolonged length of stay, increased medical costs, and increased postoperative mortality (Jankowski et al., 2011; Mouchoux et al., 2011; Veiga et al., 2012). POD typically occurs within hours or days, with fluctuating conditions, and its symptoms may often appear, worsen, alleviate, or disappear within 24 h, with significant volatility (Rudolph & Marcantonio, 2011; van Meenen et al., 2014). There is thought to be a high rate of missed diagnosis of POD that in some situations may be higher than 60% (Kishi et al., 2007). The confusion assessment method (CAM) is a simple, practical, and reliable method developed for evaluating POD in inpatients. Although there are some limitations in the diagnosis of some cases, it can be used to complete the clinical diagnosis of delirium in five minutes with a diagnosis rate of 84–100%, which means that it is widely applied (Rapp et al., 2000).

Studies have shown that the application of electroencephalogram (EEG) monitoring, such as bispectral index (BIS), can reduce the incidence of POD (Whitlock et al., 2014). Perianesthesia sedatives, especially benzodiazepines, can increase the clinical incidence of POD (Pandharipande et al., 2006). However, whether the use of opioid analgesics and the intravenous anesthetic propofol influences POD remains controversial (Brown et al., 2013; Radtke et al., 2008). Moreover, it is unclear whether the incidence of POD in elderly patients with hip fractures is affected by these factors. The risk factors for delirium may also vary depending on the individual condition and type of surgery. There have been few studies into risk factors for POD after hip surgery (Guo et al., 2016; Wang et al., 2018), but no risk stratification based on these factors has been available.

Therefore, this study was designed in order to investigate the incidence and risk factors of POD in elderly patients with hip fractures. A risk stratification index (RSI) model was then established based on the weighed odd ratio (OR) of different variables so as to provide a theoretical basis for the early prevention of POD.

## 2 MATERIALS AND METHODS

### 2.1 Study population and design

This was a retrospective study, which included elderly patients with hip fractures (femoral neck, intertrochanteric, and subtrochanteric fractures) who were no less than 65 years old and had been scheduled for surgery at the Department of Orthopedics in Peking University Shougang Hospital Hospital from March 2018 to December 2019 according to a Current Procedural Terminology (CPT) code (S72.001; S72.104; S72.201). Patients who had depression, preoperative delirium, diagnosed dementia, or other cognitive dysfunction and those with incomplete clinical information were excluded.

This study received institutional review board approval. The chart review of the cohort of 272 patients was covered by a hospital quality and improvement project, and patient consent was waived.

### 2.2 Data collection

General anesthesia (including that combined with nerve block) and combined spinal and epidural anesthesia (CSEA) were performed. For intravenous‐inhalation combined anesthesia, venous access was opened after entering the room. Routine electrocardiogram, noninvasive blood pressure, pulse oxygen saturation, and body temperature monitoring were performed. Sufentanil, rocuronium bromide, etomidate, and propofol were used for the sequential induction of general anesthesia. After the muscle relaxation was complete, endotracheal intubation was performed. Then, 1−2% sevoflurane was inhaled, supplemented by continuous intravenous propofol 4–6 mg/kg/h and remifentanil hydrochloride 4–8 μg/kg/h. For CSEA, 0.75% large‐dose ropivacaine hydrochloride was injected into the subarachnoid space of L2‐3 or L3‐4, and the highest level was adjusted to not exceed T4. Appropriate sedative drugs were given. BIS monitoring was performed, and the intraoperative anesthesia depth was maintained at 40–60. The conditions of the patients were observed and recorded by a full‐time visiting doctor or nurse at 4:00 PM once daily for five consecutive days after surgery.

Factors related to the perioperative period and postoperative outcomes were recorded in detail, including (1) general conditions: American Society of Anesthesiologists (ASA) physical status classification, gender, age, height, weight, and education background; (2) preoperative factors: comorbidities (coronary heart disease, hypertension, diabetes, pulmonary infection, history of cerebrovascular disease, emphysema, pulmonary heart disease, etc.) and drinking history; (3) intraoperative factors: type of surgery (emergency or not), type of anesthesia, surgery duration, application of BIS, intraoperative hemoglobin level from blood gas analyzer, intraoperative lactic acid measurement, total infusion, intraoperative blood loss, and transfusion; (4) postoperative factors: visual analog scale (VAS) for pain 24 h after surgery; (5) delirium situation: occurrence of event, duration, and clinical manifestations; and (6) hospitalization: length of stay, cost of hospitalization, and outcome of the patients. The VAS score was assessed within 24 h after surgery, during the 16:00 round. The VAS was evaluated by a doctor or a nurse with uniform training.

### 2.3 Diagnostic criteria

With reference to the CAM standards developed by the American Psychiatric Association, the diagnostic criteria were the following (Wei et al., 2010): (1) acute onset and fluctuating conditions; (2) inattention; (3) disordered thinking; and (4) changes in consciousness level, delirium could be diagnosed by the presence of (1) and (2), plus either of (3) or (4).

### 2.4 Sample size

We first conducted a chart review from January 2018 to March 2018, which included 16 hip fracture patients with 3 postoperative delirium (POD) (18.75%). Therefore, according to the incidence of POD reported in available studies and a pretrial study (van Meenen et al., 2014), it was assumed that the expected positive rate *π* is 20.0%, the relative error ε is 20%, the allowable error E is 5%, and the confidence level 1 – *α* is 95%, so the sample size was calculated as 246 cases.

$$n\approx \frac{{\left(Z_{\alpha /1}\right)}^{2}\pi \left(1-\pi \right)}{E^{2}}$$

### 2.5 Statistical methods

Stata 15.0 (StataCorp LP, College Station, TX, USA) statistical software was used for the analysis. Measurement data were denoted as $\bar{x}\pm$ standard deviation (SD), and comparisons between groups were performed using *t*‐test. Count data were denoted as sample rate or composition ratio, and comparisons between groups were performed using the *χ* ^2^ test. For an analysis of risk factors, multifactor backward stepwise logistic regression analysis was used in order to screen out the independent risk factors (excluding variables with *p* > .05). And to avoid multicollinearity between variables, those with VIF > 2 were excluded. All included variables were dichotomous. The weighted RSI model was established according to the odds ratio (OR) of the corresponding independent risk factors. *p* < .05 was considered statistically significant.

## 3 RESULTS

### 3.1 Patient demographic information and risk factors related to delirium

This was an open study with no restrictions on various treatment measures throughout the perioperative period. Patients were excluded due to incomplete information and compliance with exclusion criteria (*n* = 11). Finally, all of the 272 patients who remained in the study underwent hip fracture surgery.

Among the 272 elderly patients undergoing hip fracture surgery, a total of 52 patients had POD (19.12%). The patients were divided into a POD group and a non‐POD group based on whether or not POD had occurred. A univariate analysis was performed on all patients. Compared with the non‐POD group, elderly patients undergoing hip fracture surgery in the POD group showed statistically significant differences in the following variables (*p* < .05), including age (≥85), ASA classification (>II), type of anesthesia (general anesthesia), surgery duration (>120 min), perioperative infusion, intraoperative hemoglobin level (Hb < 8 g/dl), perioperative lactic acid measurement (Lac > 2 mmol/L), preoperative pulmonary, cardiovascular diseases, diabetes and drinking history (> 3 times/week), and postoperative VAS scores (Table 1).

**TABLE 1:** Risk factors for delirium in the POD and non‐POD groups

Risk factors	POD group *N* = 52	Non‐POD group *N* = 220	*p* Value
Age (>85 years), *n* (%)	27 (51.92%)	157 (71.36%)	.001
Gender, *n* (%)			.976
Male	15 (28.85%)	63 (28.64%)	
Female	37 (71.15%)	157 (71.36%)	
BMI, mean ± SD, kg/m^2^	23.56 ± 3.22	23.73 ± 3.04	.718
ASA classification, *n* (%)			<.001
Level II	9 (17.31%)	126 (57.27%)	
Level III + IV	43 (82.69%)	94 (42.73%)	
Education level, *n* (%)			.586
University	15 (28.85%)	58 (26.36%)	
Nonuniversity	37 (71.15%)	162 (73.64%)	
Drinking history (>3 times/week), *n* (%)	30 (57.69%)	65 (29.55%)	<.001
Admission method (emergency), *n* (%)	8 (15.38%)	20 (9.09%)	.179
Preoperative complications, *n* (%)			
Pulmonary disease	27 (51.92%)	45 (20.45%)	<.001
CVD	15 (28.85%)	29 (13.18%)	.006
Diabetes mellitus	25 (48.08%)	41 (18.64%)	.000
Cerebrovascular disease	6 (11.54%)	23 (10.45%)	.820
Intraoperative factors, *n* (%)			
Type of anesthesia			.023
General anesthesia	25 (48.08%)	69 (31.36%)	
Intraspinal anesthesia	27 (51.92%)	151 (68.64%)	
Surgery duration (>120 min), *n* (%)	47 (90.38%)	156 (70.91%)	.004
Infusion volume (>1000 ml), *n* (%)	39 (75.00%)	150 (68.18%)	.337
BIS	13 (25%)	76 (34.55%)	.187
Blood transfusion	27 (51.92%)	116 (52.73%)	.917
Hemoglobin level (<8 g/dl), *n* (%)	13 (25%)	37 (16.82%)	.171
Lactic acid (>2 mmol/L), *n* (%)	32 (61.54%)	65 (29.55%)	.000
Blood loss volume, mean ± SD, ml	210.19 ± 236.22	212.02 ± 291.72	.967
Postoperative factors, *n* (%)			
VAS score (>3)	31 (59.62%)	32 (14.55%)	<.001

Abbreviation: POD = postoperative delirium, BMI = body mass index, ASA = American Society of Anesthesiologists, CVD = cardiovascular disease, BIS = bispectral index, VAS = visual analogue score.

### 3.2 Risk factors independent of delirium

Using delirium occurrence as the dependent variable, a multifactor stepwise logistic regression analysis was performed (excluding variables with *p* > .05 from univariate analysis). The results showed that drinking history (>3 times/week), intraoperative lactic acid measurement (>2 mmol/L), postoperative VAS scores (>3), ASA > Ⅱ, and preoperative diabetes were independent risk factors of POD in elderly patients undergoing hip fracture surgery, while the intraoperative application of BIS was a protective factor (Table 2).

**TABLE 2:** Independent risk factors of postoperative delirium and their weighting in the risk stratification

Risk factors	OR	95% CI	*p* Value	Weighted OR
Intraoperative lactic acid (>2 mmol/L)	4.60	1.907–11.106	.001	1
Preoperative diabetes	7.51	2.903–19.416	.000	1
BIS	0.16	0.053–0.498	.001	−1
ASA level (>II)	11.09	3.817–32.201	.000	2
VAS scores (>3)	19.22	6.446–57.328	.000	2
Drinking history (>3 times/week)	3.13	1.293–7.557	.011	1

Abbreviation: CI = confidence interval, OR = odds ratio, ASA = American Society of Anesthesiologists, BIS = bispectral index, VAS = visual analogue score.

### 3.3 Establishment of a risk stratification index

**FIGURE 1:**
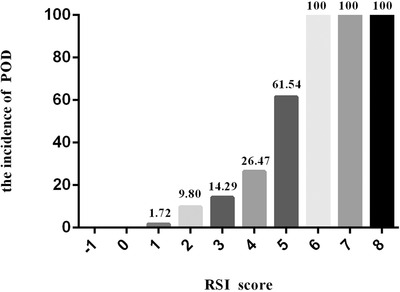
Graph showing the incidence of postoperative delirium (POD) in patients graded according to their risk stratification index (RSI) score

According to the odds ratio (OR) of the corresponding independent risk factors shown in Table 2, we established a weighted RSI model. In order to simplify the model for clinical use, we assigned a weight of 1, 2, and 3, corresponding to OR of 1–10, >10–20, and >20, respectively. If OR < 1, then it was converted to 1/OR (1/applied BIS = 6.25) and weight OR = −1 (protection factor). So, the RSI of POD in elderly patients undergoing hip fracture surgery resulted in a maximum score of 8. When the RSI was −1 to 8, the incidences of POD in elderly patients with hip fracture were 0%, 0%, 1.72%, 9.80%, 14.29%, 26.47%, 61.54%, 100%, 100%, and 100%, respectively. Figure 1 shows the incidences of POD in elderly patients undergoing hip fracture surgery according to different RSI values. When the RSI ≥ 5, the rate of postoperative delirium significantly increased (*p *< .05, Figure 1).

### 3.4 Clinical prognosis of patients

Compared with the non‐POD group, the POD group showed a significant increase in the average length of hospital stay (*p* < .05) and a moderate increase in medical costs and mortality (Table 3).

**TABLE 3:** Comparison of outcomes in the two groups

	POD *N* = 52	Non‐POD *N* = 220	*p* Value
Hospital stay (d) mean ± SD	12.94 ± 2.70	11.98 ± 3.19	.004
Medical cost (ten thousand) mean ± SD	8.84 ± 3.98	8.24 ± 3.12	.239
Mortality (*n*, %)	1 (1.92%)	3 (1.36%)	.763

## 4 DISCUSSION

The aim of this study was to investigate the incidence of POD in elderly patients who underwent hip surgery and to ascertain factors that increase the risk of POD. This information was then used to develop an RSI system that may be able to identify patients at risk of POD. The results show that POD occurred in 19.12% of patients. Independent risk factors for POD included drinking history (> 3/ week), the perioperative lactic acid level (Lac > 2 mmol/L), postoperative VAS, ASA physical status > II, application of the BIS index, and preoperative diabetes. These factors were weighted and used in the RSI, which gave a maximum score of 8. Patients with an RSI score ≥5 had a significantly increased risk of POD.

To date, there have been few studies that have concentrated on risk factors for POD after hip fracture. One previous study found older age, lower albumin, a history of stroke, higher blood glucose, higher total bilirubin, higher C‐reactive protein, longer duration of surgery, and a higher volume of red blood cell transfusions were independent risk factors of POD in elderly patients following total hip arthroplasty for hip fracture (Guo et al., 2016). While another study found that age over 75 years old, diabetes, and ASA classification > II were independent risk factors for POD (Wang et al., 2018). Our results show some differences with both these studies. Diabetes or higher blood glucose were identified in both the studies in agreement with our results, and ASA > 2 was identified in one of the studies. However, age was not identified as an independent risk factor. While preoperative drinking, perioperative lactic acid level > 2 mmol/L, postoperative VAS, and application of BIS that were identified in this study were not identified in the previous studies. Differences between the studies highlight the need for larger multicenter studies to evaluate risk factors for POD after hip surgery.

The risk factors identified by this and prior studies suggest three major contributors for the occurrence of POD: underlying brain health (age, drinking, and diabetes), the effects of specific medications and anesthetics (propofol and general anesthesia), and clinical distress during and after surgery (lactic acid and VAS scores). Each of these contributors will be discussed in turn.

The age limit of susceptibility to delirium remains controversial. The statistical results of the elderly patients in this study found that the age of >85 years old is not an independent risk factor, and the number of patients aged >85 years old in the delirium group is less than that in the non‐POD group (51.92% < 71.36%). However, it is generally believed that elderly patients are the high‐risk group for POD (Elie et al., 1998; Winkler et al., 2011). In addition to age, long‐term drinking can also cause toxic reactions to multiple regions in the cerebral nervous system, especially the prefrontal lobe, resulting in amnestic cognitive impairment. This study showed that patients with a long‐term drinking habit (≥3 times/week) are more likely to have delirium after surgery (Dickov et al., 2012). The elderly patients undergoing hip fracture surgery can be subjected to complicated perioperative conditions, greater surgical trauma, and stronger stress response. Preoperative ASA classification is a preliminary assessment of the patient's tolerance to anesthesia, and it can also basically reflect the physical status of the patient. In this study, preoperative pulmonary, cerebrovascular, and cardiovascular diseases were excluded from the independent risk factors. However, preoperative diabetes was an independent risk factor for POD in elderly patients undergoing hip fracture surgery may be caused by the impaired cerebral blood flow due to the involvement of the cerebral and carotid arteries, as well as the autoregulation that affects cerebral blood flow (Gummert et al., 2002).

The impact of type of type of anesthesia on POD is still being debated (Mason et al., 2010). In this study, two types of type of anesthesia, intravenous‐inhalation combined anesthesia versus CSEA, were not independent risk factors of POD. Nevertheless, compared with intravenous inhalation combined anesthesia, CSEA could reduce the incidence of POD in elderly patients with hip fractures by 11.4% (Table 1). As for the underlying reason, the role of anesthetics is inconclusive (Chandler et al., 2013). Nishikawa holds that propofol promotes the occurrence of POD through drug redistribution in the body leading to peripheral drug reflux that affects the mental function of patients (Nishikawa et al., 2004), while Gerretsen believes that it works by blocking muscarinic acetylcholine receptors (mAChRs) (Gerretsen & Pollock, 2011). For inhaled anesthetics, Meyer found that both isoflurane and sevoflurane could promote the occurrence of POD (Meyer et al., 2007). The occurrence of POD is generally believed to be associated with intraoperative hypotension, postoperative hypoxemia, and postoperative pain (Cole, 2014; Leung et al., 2009; Wang et al., 2015). Although all patients in this study were given sufficient analgesia during the surgery, given the varying conditions and individual differences, the patients with VAS > 3 were more likely to develop POD. Due to the particularity of elderly patients undergoing hip fracture surgery, the intraoperative measurement of lactic acid can be used to directly assess whether or not a patient is suffering from hypoxia and lactic acidosis.

A potential application of this study is to prospectively predict POD in hip fracture patients. Identifying high‐risk patients and conducting early intervention should decrease the incidence of POD. It is of clinical importance since POD is a serious complication that can lead to the worsening of patients’ prognosis due to intolerance to tracheal intubation, a prolonged length of stay, increased medical costs, and increased postoperative mortality (Jankowski et al., 2011; Mouchoux et al., 2011; Veiga et al., 2012).

Of course, this study has some limitations. The retrospective nature of the study may convey some bias, and importantly limits the scope of the research to risk factor identification rather than clinical prediction. We hope to apply the risk factors identified here in future prospective studies to verify their predictive value. Additionally, the study was undertaken in a single center, so larger, multiple‐center studies are needed to support these results. There are also some other points that need consideration as follows: (1) Body temperature was not included in the observation factors, and its effect on POD could not be determined. At present, various thermal insulation measures throughout the perioperative period makes the guiding role of body temperature decline, while drastic changes of body temperature may increase the incidence of cognitive impairment. (2) As an important indicator of perianesthesia in‐depth monitoring, the application of BIS has been shown to reduce the incidence of POD, which is similar to this study. But its mechanisms have not been fully explored. (3) Patients with pre‐existing cognitive dysfunctions, including preoperative delirium and dementia, were excluded from the sample to eliminate the potentially confounding effects of these conditions in the diagnosis of POD, consistent with criteria in similar studies, yet it is possible that these exclusions may limit generalizability of the results to typical hip fracture patients.

In summary, we found that the incidence of POD in elderly patients undergoing hip fracture surgery was 19.12%. Stepwise logistic regression analysis showed that drinking > 3 times/week, intraoperative measurement of lactic acid > 2 mmol/L, preoperative diabetes, intraoperative application of BIS, ASA > II, and VAS > 2 were independent factors for the occurrence of POD. Considering the worsening prognosis of POD patients, such as extended cognitive impairment, prolonged length of stay, increased medical costs, and increased postoperative mortality, it is necessary to pay attention to whether adjusting the controllable factors during the perianesthesia period can affect the clinical prognosis of elderly patients undergoing hip fracture surgery. The RSI system may represent a safe and reliable guide for predicting outcomes of elderly patients with hip fractures, such that RSI ≥ 5 may predict the onset of postoperative delirium.

## FUNDING

This study was supported by Wu Jieping Medical Foundation (320.6750.19018) and Peking University Shougang Hospital Scientific Research and Development Funds (2019‐yuan‐lc‐10).

## CONFLICT OF INTEREST

All authors declare that they have no conflict of Interest.

### PEER REVIEW

The peer review history for this article is available at https://publons.com/publon/10.1002/brb3.2420
